# The efficacy of pre-emptive analgesia on pain management in total knee arthroplasty: a mini-review

**DOI:** 10.1186/s42836-019-0011-7

**Published:** 2019-10-22

**Authors:** Jianda Xu, Huan Li, Chong Zheng, Bin Wang, Pengfei Shen, Zikang Xie, Yuxing Qu

**Affiliations:** 1 Department of Orthopaedics, Changzhou Traditional Chinese Medical Hospital, Nanjing University of Traditional Chinese Medicine, 25 North Heping Road, Changzhou, 213000 Jiangsu Province China; 2 Department of Arthroplasty, The First People’s Hospital of Changzhou, Changzhou, 213003 China

**Keywords:** Pre-emptive analgesia, Pain management, Total knee arthroplasty, Mechanism, Methods

## Abstract

Total knee arthroplasty (TKA) is considered a cost-effective and efficacious treatment for patients with end-stage knee arthritis. Meanwhile, TKA has been regarded as one of the most painful orthopaedic surgeries. Pain control after TKA remains a challenging task. Many analgesic innovations are used to reduce the level of pain, but none has been proven to be the optimum choice till now. Multimodal analgesia incorporates the use of analgesic adjuncts with different mechanisms of action to enhance postoperative pain management. This approach is a preferable choice in relieving postoperative pain with minimum side effects. This paper aims to review pre-emptive analgesia for pain management in TKA. We reviewed the application of pre-emptive analgesia, its physiological mechanism, and the techniques.

## Introduction

Total knee arthroplasty (TKA) is considered a cost-effective and efficacious treatment for patients with end stage knee arthritis [1]. Meanwhile, TKA has been regarded as one of the most painful orthopaedic surgeries [2]. Pain can impact significantly upon patient’s rehabilitation, as well as physical, emotional and social wellbeing [3]. Many analgesic innovations (such as intravenous patient-controlled analgesia, peripheral nerve blockade, and continuous epidural analgesic techniques) are used to reduce the level of pain, but none has been proven to be the optimum choice till now [4].

Preemptive analgesia (PA) is defined as an anti-nociceptive treatment that prevents the establishment of altered central processing of afferent input, which amplifies pain after TKA [5]. The idea of PA was first proposed by Crile [6] in 1913 and disseminated by Wall and Woolf [7]. Woolf [8] reported that PA could change the time and magnitude of pain postoperatively by decreasing central sensory processing. Bach et al. [9] in 1988 found that 3 days of continuous epidural morphine plus bupivacaine administered prior to amputation reduced the incidence of phantom limb pain 6 months later compared with a control group that did not receive an epidural before amputation. In an experimental study, Coderre et al. [10] found PA prevented the nervous system from experiencing pain from the surgery and thereby reduced postoperative pain as well.

PA focuses on postoperative pain control and the prevention of central sensitization and chronic neuropathic pain by providing analgesia administered preoperatively but not after surgical incision [11]. PA has protective effect on the nociceptive system, which could significantly reduce the level of pain and decrease the risk for the development of chronic pain [8]. Because the pain mediators in peripheral and central nervous systems are inhibited, the duration and level of postoperative pain is decreased. The benefits from PA will outlast their normal pharmacological duration of action.

Multimodal analgesia incorporates the use of analgesic adjuncts with different mechanisms of action to enhance postoperative pain management [12]. This approach is a preferable choice in relieving postoperative pain with minimium side effects. Skinner et al. [13] found that PA with multiple non-narcotic medications used in a stacked modality could significantly reduce postoperative pain. PA is an important part of multimodal analgesia.

This paper aims to review PA for pain management in TKA. Three questions are raised as a framework of this review:

1. Why should we care about PA?
2. What’s the physiological mechanism of PA?
3. What are the techniques of PA?

## Why should we care about PA?

Compared with a similar analgesic treatment after TKA, PA improves the pain relief apparently [10]. Shlaifer et al. [11] found that preemptive periacetabular bupivacaine provided a better postoperative pain relief than intra-articular injection. Gottchalk et al. [12] advocated that preemptive epidural analgesia significantly attenuated postoperative pain during hospitalization and even after hospital discharge for a long time.

The tissue noxious stimuli activate pain that often launches a cascade of alternative reactions, which brings a central and peripheral sensitization. Both peripheral sensitization (with a reduction in the threshold) and central sensitization (with excitability of spinal neurons) contribute to hypersensitivity state of injury pain [8]. Peripheral sensitization usually has a lower threshold, while central sensitization brings hyper-excitability to uninjured tissues [9]. These alterations amplify the severity of the pain by elevating response to noxious stimuli. Therefore, pain management should include both the nociception and central nervous system related to tissue trauma. Prescribing analgesics can effectively reduce the pain after TKA, but this management is suboptimal. The new strategy is to prevent abnormal sensitization of peripheral and central neurons.

The knee pain after TKA is a sharp pain. Adequate and appropriate postoperative analgesia allows an earlier rehabilitation, resulting in better joint function. PA can achieve a better pain control, faster post-operative functional recovery, and leads to less adverse events [13]. Some researches showed that efficacy of the analgesic drugs are affected by patient’s personality and behaviors. Patients with less anxiety and depression are associated with lower levels of joint pain and lower use of opioid analgesics [14]. The patients who undergo TKA usually have a long earlier history of knee pain, associated with higher anxiety. Clearly, the persistent afferent barrage from the wound (free endings of primary afferent neurons) can induce severe acute pain postoperatively, especially at the time that spinal anaesthetic has worn off. Since the postoperative pain tends to be severe, PA has been considered to play an important role in the pain management [15]. In patients receiving total hip arthroplasty, preoperative intravenous administration of ketorolac often caused less pain and delayed the time of postoperative opioid consumption [16]. Briefly, the PA is associated with less subsequent pain postoperatively.

## What’s the physiological mechanism of PA?

The physiological mechanism of PA is complex and involves the pain pathways. The pain pathways involved in reflex response that is entirely within the spinal cord, and the withdrawal response occurs without the sensation of pain. When the pain withdrawal reflex fails to disappear long after the triggering event. Two-neuron arc is the simplest form, fastest-responding and consists of afferent and efferent neurons. One example is the knee-jerk reflex. The three-neuron arc reflex arc consists of afferent neurons, interneurons, and efferent neurons. Afferent neurons conduct impulses to the central nerve system from the receptors. Efferent neurons conduct impulses from the central nerve system to effectors (muscle or glandular tissue).

Noxious stimuli are stimuli that elicit tissue damage and activate nociceptors. Nociceptors are sensory receptors (primary afferent neurons) that detect signals from damaged tissues and indirectly also respond to chemicals released from the damaged tissues. The noxious stimuli usually produce two types of sensory inputs, i.e., tissue damaging noxious stimuli and inflammatory stimuli from algetic substances (prostaglandins, 5-HT, bradykinin, histamine, and so on) [17]. Many inflammatory mediators increase the sensitivity of nociceptors. At the same time, the peripheral terminals of nociceptors convert chemical, mechanical or thermal energy to electrical activities, which are subsequently transmitted to the dorsal horn of the central nervous system [9]. Based on the types of stimuli and their locations, nociceptors are subdivided into several groups, including myelinated Aδ-fiber nociceptors for mechanical and thermal injuries; unmyelinated C nociceptors for strong mechanical, thermal and/or chemical stimuli. The Aδ-fiber nociceptors mediate the ‘first pain’ (rapid, sharp pain), while C nociceptors mediate ‘second pain’ (delayed pain).

The secondary nociceptive neurons in the dorsal horn receive the pain signals from nociceptors, which respond for further transmission of pain sensation. These nociceptive neurons include nociceptive-specific neurons (just for signals in Aδ-fiber nociceptors) and wide-dynamic-range neurons (for signals in Aδ-fiber and C-fiber nociceptors and non-nociceptive impulses in Aβ-fiber nociceptors). The activity of these secondary nociceptive involves the balance between excitatory and inhibitory factors from different transmitted elements.

If the severity of injuries is below the threshold of tolerance, there exists a consistent and proportionate relationship between the stimulus and pain reaction. Whereas, the damage will initiate an alternative transmission process. The central and peripheral sensitization are induced. The touch signals from Aβ-fiber nociceptors will be recognized as pain signals by wide-dynamic-range neurons. The pain-promoting substances from the free endings of primary afferent neurons and extraneural sources result in a peripheral sensitization. The signals from Aδ-fiber and C-fiber nociceptors will be amplified, which brings pain sensation due to a lower damage. A memory trace of pain emerges with a prolonged alternative pain reaction [18].

Therefore, pre-emptive measures interrupting these alternative processes are needed for better analgesia. PA is an effective supplement to multimodal analgesia approach in relieving postoperative pain. The combined approach may contribute eventually to a longer post-operative painless course, earlier mobilization, and better knee joint function [19].

## What are the techniques of PA?

Given the importance of PA, we tried many different analgesics or analgesic interventions. These interventions direct at the periphery, and transmit along the afferent/efferent pathways and central neurons [20]. These analgesia strategies can also interfere with one or more sites along the pain pathway. These trials include nonsteroidal anti-inflammatory drugs (NSAIDs), opioids, N-methyl-D-aspartate receptor antagonists, peripheral local anesthetics, and systemic antiepileptics.

### NSAIDs

NSAIDs, as important pain killers, have antipyretic actions with less postoperative side effects compared with opioids [21]. The nonselective NSAIDs inhibit cycloxygenase (COX) enzymes covering COX-1 and COX-2 enzymes. Considering the disturbance of normal platelet function and gastrointestinal toxicity by inhibiting COX-1 enzyme, the selective COX-2 inhibitors offer a better choice for perioperative application. Some researcher suggested that preoperative administration of NSAIDs could prevent the secondary prostaglandin hyperalgesia. The prostaglandin and bradykinin releases are the main causes of inflammatory pain, which alter nociceptive sensitivity and sensitivity threshold [22].

In orthopedic surgeries, NSAIDs have been widely used to reduce postoperative pain. Kashefi et al. [23] reported that preoperative oral celecoxib at the dose of 200 mg every 2 h could effectively control the postoperative pain in patients who underwent lower extremity orthopedic surgeries. Compared with celecoxib and placebo, etoricoxib was more effective for PA in controlling postoperative pain [24]. A new research proposed that, in patients who underwent TKA, PA improved the analgestic effect of multimodal analgesic regime, reduced inflammatory reaction, and accelerated functional recovery during the first postoperative week [25]. However, PA didn't further improve long-term function of the knee. O’Hanlon et al. [26] suggested tenoxicam should be given intramuscularly 30 min preoperatively. The striking advantages were the lower total doses of demerol and diclofenac, and the significantly extended time to first analgesic requirements.

In controlling thermal hyperalgesia, NSAIDs have a comparable antagonist effect on N-methyl-D-aspartate receptor and serve as useful analgesic adjuncts to the control of acute pain [27]. In addition to the central mechanism, NSAIDs can also contribute to the prevention of spinal prostanoid synthesis by reducing release of neurotransmitters from reflex arc of pain [28]. The effect of PA is caused by their actions of inhibiting the synthesis of prostaglandins through the inactivation of cyclooxygenase.

There are some contradicting and even negative results. By comparing combined pre- and postoperative NSAIDs and post-operative NSAIDs alone, Murphy [29] found that pre-emptive administration of indomethacin brought the equivalent postoperative analgesia and opioids consumption.

### Opioids

Opioids are mostly used analgesics for pain control and they work by interacting with opioid receptors. Some researchers pointed out that preemptive use of intrathecal morphine injection had a strong and long-lasting effect in postoperative analgesia [30]. Campiglia et al. [31] reported that sublingually-given morphine sulphate in abdominal surgery provided a better pain control over a period of 48 h postoperatively. However, for patients receiving abdominal hysterectomy, intravenous alfentanil either at induction or one minute after skin incision brought no difference in postoperative pain control [32].

Chong et al. [33] found that intravenous infusion of morphine for PA was more effective in preventing the occurrence of phantom limb pain. Preemptive opioid analgesics in orthopedic surgeries could delay the time of requesting analgesics postoperatively [34].

### N-methyl-D-aspartate receptor antagonists

There is a controversy about the role of N-methyl-D-aspartate receptor antagonists (NMDA antagonists) in PA. The extensive administration of NMDA-channel antagonists has been considered an effective way to block central sensitization. Oliveira et al. [35] blocked NMDA receptors in the neurons of dorsal horn of spina, resulting in inhibition of central sensitization triggered by nociceptive stimulations. Malmberg et al. [27] reported that spinal administration of NMDA could induce thermal hyperalgesia. However, a meta-analysis concluded that pre-emptive ketamine was less effective in controlling postoperative pain [18]. Li et al. [36] found that preemptive administration of dextromethorphan 30 min before operation significantly reduced postoperative fentanyl consumption and the incidence of nausea and vomiting in the patients given patient-controlled intravenous analgesia. In patients with TKA, Yeh et al. [37] failed to observe any PA effect of dextromethorphan on postoperative pain in patients under epidural anesthesia. However, dextromethorphan given either before or after surgery augmented the effect of another analgesic (morphine) and thereby achieved a better pain relief.

Up to now, the clinical efficacy of NMDA antagonists in PA remains controversial.

### Peripheral local anesthetics

Local peripheral anesthetics inhibit pain transmission by stabilizing the cell membrane and controlling the sodium influx [38]. One research found that local PA couldn't improve the level of pain relief compared with a similar post-incisional infiltration [39, 40]. However, many studies still argued that preincisional infiltration with local anesthetics produced a better pain relief compared with either spinal anesthesia or general anesthesia alone. Lower level of pain would last a few more days [41]. Joel et al. [42] proposed that rectal administration of PA brought an unexpected benefit and reduced postoperative dose of opioids. PA was a key approach for postoperative analgesia. Gordon et al. [43] proposed that administration of bupivacaine (a long-acting local anesthetic agent) could block the peripheral nociceptive input and the development of central hyperexcitability. The analgesia brought less pain and analgesic consumption. A study about preemptive epidural analgesia in laparoscopic radical hysterectomy for cervical cancer revealed that preemptive epidural analgesia could prevent postoperative pain and control cytokine response [43]. Dahl-V et al. [44] reported that pre-incisional infiltration with local anaesthetics had a tendency toward faster awakening, earlier recovery, and less opioid use postoperatively. Compared with the administration at the end of surgery, preemptive intra-articular injection of morphine and bupivacaine provided a longer analgesia time [45].

### Systemic antiepileptics

Systemic antiepileptics are GABA analogues that are originally used as therapeutic agent for partial seizures. The central and peripheral sensitization are considered to be an important mechanism of postoperative pain [46]. GABA analogues can effectively suppress the hyperalgesia of dorsal horn neurons. The mechanism is that gabapentin and pregabalin can bind to alpha-2 delta subunit of voltage-gated calcium channels to induce their analgesic action. On binding to alpha-2 delta subunit, pregabalin was six times more potent than gabapentin with less adverse events [47]. A review showed that gabapentin and pregabalin used as PA could effectively reduce postoperative analgesic rescue and delay the first time of analgesic requirement [48]. Gabapentin reduces the opioid consumption at the first 24 h postoperatively, but the effect is not dose-depenent. The highest safe doses of gabapentin and pregabalin are 1200 mg and 300 mg, respectively. Saraswat [49] proposed that the first time of analgesic requirement in gabapentin group was earlier than in group.

## Conclusion

Always, prevention is preferable to cure. PA is a good supplement to multimodal approaches, and is confirmed to be a better analgesic choice that prevents central sensitization in multiple sites along the pain pathways. Although most studies reported that various agents/techniques used for PA have potential in controlling postoperative pain, none of them has obvious superiority. Negative clinical findings are still not uncommon. On the basis of better understanding of pain mechanism, choosing a suitable analgesic approach (either alone or in combination) is essential for postoperative pain control. In PA, we should focus on attenuating the impact of the noxious stimuli in advance, but not just the time.

Till now, many questions remain unanswered about PA: Which approach is the best? What is the optimal dose to suppress the processes of peripheral and central sensitization? What’s the necessity to extend the time of preemptive analgesia to postoperative period for reserving the initial advantage?

Most positive clinical and experimental results indicated that the pre-emptive could reduce postoperative pain. However, achieving the maximal analgesic effect of PA is still a challenge. Futher studies are needed to find a better comprehensive approach.

## Data Availability

Data sharing not applicable to this review as no datasets were generated or analysed during this review.

## Abbreviations

COX: Cycloxygenase enzymes
NMDA antagonists: N-methyl-D-aspartate receptor antagonists
NSAIDs: Nonsteroidal anti-inflammatory drugs
TKA: Total knee arthroplasty
γ-aminobutyric acid analogues: GABA analogues
