# Correction to: The efficacy of pre-emptive analgesia on pain management in total knee arthroplasty: a mini-review

**DOI:** 10.1186/s42836-019-0013-5

**Published:** 2019-11-05

**Authors:** Jianda Xu, Huan Li, Chong Zheng, Bin Wang, Pengfei Shen, Zikang Xie, Yuxing Qu

**Affiliations:** 1 Department of Orthopaedics, Changzhou Traditional Chinese Medical Hospital, Nanjing University of Traditional Chinese Medicine, 25 North Heping Road, Changzhou, 213000 Jiangsu Province China; 2 Department of Arthroplasty, The First People’s Hospital of Changzhou, Changzhou, 213003 China

**Correction to: Arthroplasty (2019) 1:10**

**https://doi.org/10.1186/s42836-019-0011-7**

In the original publication of this article [1], Xu Jianda and Li Huan are co-first authors. This information is accidentally missed during the copyediting. In addition, the author names' first name and last name interchanged, and the correct names should be: Jianda Xu, Huan Li, Chong Zheng, Bin Wang, Pengfei Shen, Zikang Xie, and Yuxing Qu.   

The original article has been corrected for the changes. 

## References

[1] Xu J, et al. The efficacy of pre-emptive analgesia on pain management in total knee arthroplasty: a mini-review. Arthroplasty. 2019;1:10. 10.1186/s42836-019-0011-7. 10.1186/s42836-019-0011-7 PMC8796433 35240765

